# Interactive van Krevelen diagrams – Advanced visualisation of mass spectrometry data of complex mixtures

**DOI:** 10.1002/rcm.7823

**Published:** 2017-02-20

**Authors:** William Kew, John W.T. Blackburn, David J. Clarke, Dušan Uhrín

**Affiliations:** ^1^ EaStCHEM, School of ChemistryUniversity of EdinburghEdinburghEH9 3FJUK

Dear Editor,

The field of complex mixture analysis has advanced significantly in the past two decades, although its history goes much further back. When Dirk Willem van Krevelen developed his now eponymous diagram in 1950 to represent the chemical makeup of coals, he proposed that the chemical nature of samples, including the presence of structural motifs and chemical properties, could be inferred from the elemental ratios of the sample.^1^ While his work, limited by the technology of the era, looked at whole samples characterised by the ratio of elements present, i.e. number of carbons‐to‐hydrogens within the sample, modern mass spectrometry allows us to examine in a similar manner the individual components of a complex mixture.

Since 2003, when the modern van Krevelen diagram was first used to visualise complex MS datasets,^2^ every significant high‐resolution mass spectrometric analysis of a complex mixture has included one.^3^, ^4^, ^5^ Today's van Krevelen diagram places every assigned unique chemical formula on a 2D scatter plot of H/C ratio versus O/C ratio, although other elemental ratios can also be used. Although this represents a break from the original intentions of van Krevelen, the modified technique has become a useful tool for the interpretation and visualisation of complex data. For example, regions of the van Krevelen plot can be tentatively associated with certain compound classes,^2^, ^6^, ^7^ such as lipids (O/C < 0.2, H/C 2 – values quoted are approximate), carbohydrates (H/C 2, O/C 1), or condensed hydrocarbons (O/C < 0.2, H/C < 1).

In the field of complex mixture analysis, a number of methods are available to the enterprising chemist; however, Fourier transform ion cyclotron resonance mass spectrometry (FTICR MS) reigns supreme as the ‘gold standard’ technique.^8^, ^9^ Likewise, there exist a number of well‐studied complex mixtures, including natural organic matter (NOM), i.e. dissolved organic matter,^5^, ^10^, ^11^ soil organic matter,^12^ and organic aerosols,^13^, ^14^, ^15^ petroleum,^16^, ^17^ or beverages such as wine^18^ or Scotch whisky.^19^, ^20^ Amongst the most complex of these, a component of NOM and the closest sample to a universal standard, is Suwannee River Fulvic Acid (SRFA) produced by the International Humic Substance Society.^21^ A typical electrospray ionisation (ESI)‐FTICR mass spectrum of SRFA will contain thousands of peaks across a range of masses, predominantly between *m/z* 200 and 700. Due to its ubiquity and complexity, SRFA was chosen to demonstrate the capability of the visualisation tools described herein.

With the mass accuracy of FTICR MS spectra in parts‐per‐billion,^22^, ^23^ routine and confident assignment of thousands of unique chemical formulae to individual peaks is now increasingly possible. The generation of this volume of data represents a significant challenge in terms of data visualisation, interrogation, and interpretation that has not been addressed so far. Here, we present a handful of tools aimed at filling this gap.

We have developed a version of the van Krevelen diagram, which introduces interactivity, and allows the analyst, or reviewer, to interrogate the data in an intuitive way. This interactive van Krevelen, or *i‐*van Krevelen for short, is generated using the Bokeh Python plotting library.^24^ The developed tools are fully compatible with data assigned using any software package, as the input for the *i‐*van Krevelen scripts are three text files containing (1) monoisotopic peak assignments, (2) isotopologue peak assignments, (3) remaining unassigned, but detected, peaks. Example input files are included with the suite of presented tools. The Bokeh API allows for the straightforward coding, in Python, of complex JavaScript (JSON) plots as HTML5 Canvas objects. The output from this tool is a standard HTML document compatible with any modern web browser such as Google Chrome, Firefox, or Internet Explorer.

The main feature of the *i‐*van Krevelen software is the generation of interactive diagrams including a centroid mass spectrum, van Krevelen, DBE vs carbon number plot and the modified Aromaticity Index vs carbon number plot.^25^ The plots are linked together, such that selecting any data points in one plot highlights those same points – i.e. unique chemical formula – in the other plots. In addition, these plots are explorable, featuring zoom and pan tools, as well as a display of the key information of each point in a hover‐tool. Finally, the data points can be used as hyperlinks – in our implementation, they link to a ChemSpider (The Royal Society of Chemistry, Cambridge, UK) search for their molecular formula.

The benefits of these features will be immediately obvious to any analytical chemist who has tried to make sense of complex static van Krevelen diagrams of complex mixtures.

For example, in a standard van Krevelen plot, numerous points may be superimposed if they share elemental ratios but differ in molecular formulae. As a van Krevelen plot is a specific type of scatter plot, it is susceptible to the same problems as other any other scatter plot, and can be misinterpreted when hundreds or thousands of points are plotted. Whilst the addition of colour and transparency can reduce these problems, they are not eliminated entirely.^26^, ^27^ One alternative is to plot data density, not individual data points – i.e. a histogram or kernel density plot in 1D, or a hexagonally binned data plot in 2D.^28^ This allows easier visualisation of where the most (or largest, or most intense, depending on the density variable) data points are; however, this approach leads to a loss of information about specific components and their molecular formulae. With interactivity, however, a user can zoom to a region of interest in the plot, and use the hover‐tools to identify every component contributing to a particular point, thus removing the ambiguity caused by the overlap. Furthermore, we encode the relative abundance of a species by the size of the glyph on the plot. The colour can then be used to indicate mass, as in our van Krevelen plots, or oxygen number, as in our DBE and AI plots. This approach is illustrated in our recent paper on Scotch whisky.^20^

**Figure 1:**
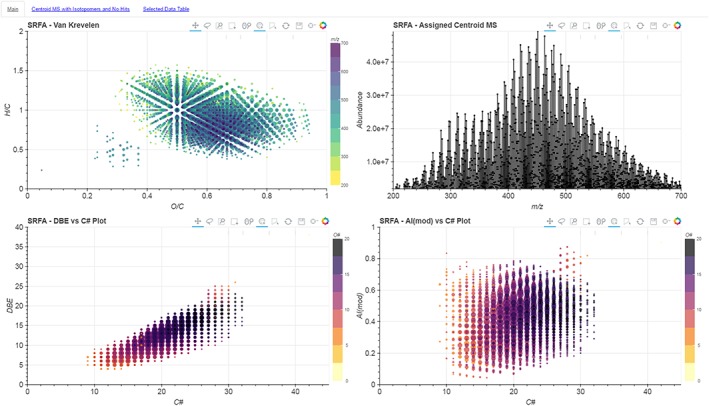
Overview screenshot of the *i*‐van Krevelen Main Page. Four sub‐plots are shown, from top left clockwise, van Krevelen, centroid mass spectrum, DBE vs C #, and AI(mod) vs C #. The scatter plots have points sized per their relative abundance, while the colour scales represent either *m/z* range (van Krevelen) or oxygen number (DBE vs C# and AI(mod) vs C#). [Colour figure can be viewed at wileyonlinelibrary.com]

**Figure 2:**
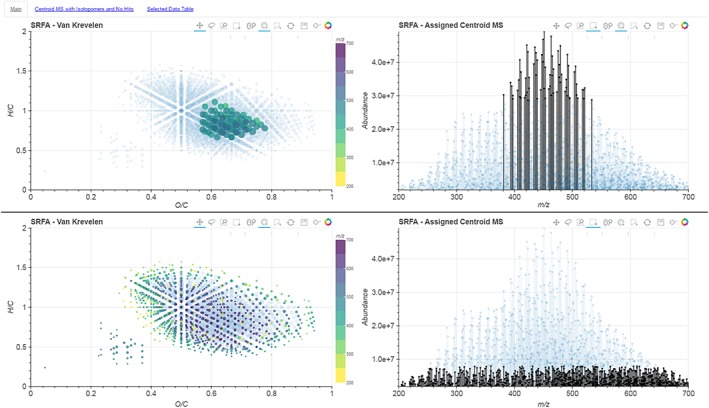
Screenshots of the van Krevelen and centroid mass spectrum plots with two different selections of data points. The top frame shows that the selection of the most abundant ions only represents a small range of chemical diversity on the van Krevelen plots, whilst the bottom frame shows that the least abundant ions, “the grass”, represent the true chemical diversity of the spectrum. [Colour figure can be viewed at wileyonlinelibrary.com]

Reducing complex data down to a two‐variable van Krevelen plot inevitably represents a loss of information. In our tool, we have therefore created several 2D plots that are linked together. An example of this layout is shown in Fig. 1. This allows for the relation of multiple variables to a single molecular formula in order to better understand the sample. For example, as shown in Fig. 2, we can select only the most intense signals in the spectrum. Here we can see that these species, whilst the dominant compounds in the mass spectrum, represent only a fraction of the diversity present in the sample as revealed by their position on the van Krevelen plot. This means that if we were to consider only the *n* most abundant ions – an approach utilised in some previous statistical analyses of complex spectra^19^ – we would be losing the vast majority of the chemical diversity of the sample. On the contrary, by selecting only the low‐abundance peaks, i.e. the “grass”, we can see that these signals do describe the chemical diversity of the sample more fully. Such information, which is lost in static van Krevelen plots, will be important for comparative studies aiming to characterise multiple samples by different ionisation techniques; for example, comparing ESI with MALDI (matrix‐assisted laser desorption/ionization) mass spectra, where the abundance of a species is a function of both concentration and ionisation energy. Likewise, this interactive selection of points can be used to easily link outliers on any plots to their positions on the mass spectrum, or understand where specific regions of these plots originate from in the mass spectra.

**Figure 3:**
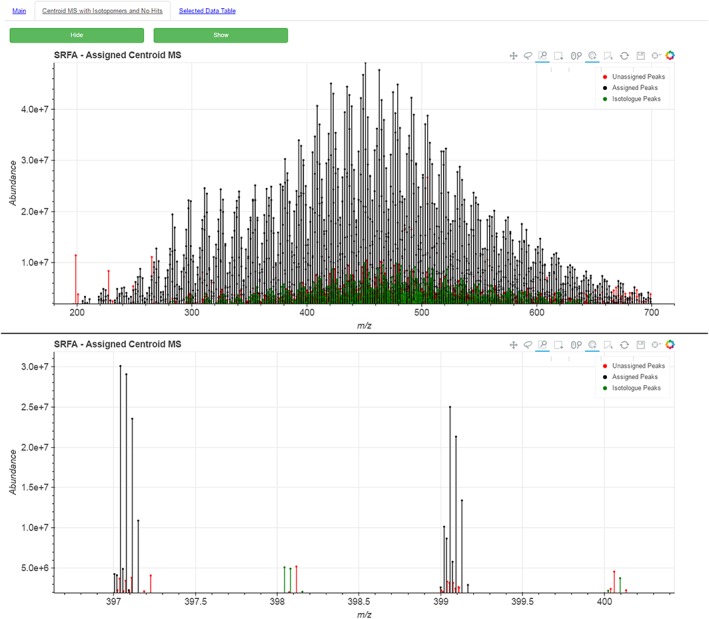
Screenshots of the centroid mass spectrum showing an overlay of peaks which represent isotopomers (green) and peaks which could not be assigned a molecular formula (red). The bottom frame shows a zoomed in region of *m/z* 397–400, clearly showing the monoisotopic peaks and their associated ^13^C isotopomers. Those peaks not assigned may be secondary isotope peaks, e.g. with two ^13^C atoms or a single ^18^O atom. [Colour figure can be viewed at wileyonlinelibrary.com]

On a second tab of the HTML page, the centroid mass spectrum is plotted with the identified isotopomers, as well as the remaining unassigned peaks. An example of this is shown in Fig. 3. This gives the analyst, and more importantly the reader or reviewer, a straightforward means to see how well the spectrum was assigned, thus validating or otherwise the assignment methodologies.

Finally, on a third tab, the data table is presented that is required to generate the plots, and it is also interactively linked to the plots, meaning that selections made on any plot are highlighted in the data table, and vice versa. This data table is downloadable as a text file.

The developed code also includes a number of related Python scripts for: (i) automated batch plotting of publication quality van Krevelen and DBE vs Carbon Number plots; (ii) heteroatomic class distribution calculation and plotting; (iii) an “all‐possible‐formula‐generator”, which calculates a list of possible, logical, chemical formulae as based on work done by Kind *et al*.;^29^ (iv) a tool to batch perform automated exact mass‐to‐formula assignment based on Kendrick mass defect analysis and z* by looking for homologous series of compounds;^30^ and (v) a tool for reformatting of PetroOrg (Florida State University, Tallahassee, FL, USA) output CSV files. Assignment files generated by the latter two tools produce, as outputs, inputs for the *i‐*van Krevelen software and other included scripts. The included formula generator is especially useful for determining assignment error thresholds, for example by allowing the user to determine the minimum distance between possible compounds at a given *m/z*, and thus adding confidence to the assignment.

Overall, these interactive plots, and their combination, represent a step forward in the analysis of complex mixtures by high‐resolution mass spectrometry. The tools are open‐source and available freely through GitHub with a GNU General Public License v3.0, encouraging others to experiment with and build upon them. The GitHub repository^31^ can be found online.^32^ An online tool allowing the use of some of these tools without the need to install any specialist software has also been developed, and can be found through the GitHub repository. An example of the interactive plots enabled by this initial *i*‐van Krevelen package based on the SRFA FTICR MS data can also be found online.^33^

Future work could incorporate the Datashader^34^ package, which would allow the visualisation of the raw profile spectra in a web browser without the need for the end user to download large data files or install proprietary mass spectrometry software, as well as the Bokeh Server tool, allowing the user to dynamically select which variables to plot on each axis, or to choose a specific colour or size scale. Examples of code for the Datashader functionality are included as a Jupyter Notebook in the GitHub repository.
